# Identification of Target Genes in Hypertension and Left Ventricular Remodeling

**DOI:** 10.1097/MD.0000000000021195

**Published:** 2020-07-10

**Authors:** Bo Pang, Cong Hu, Guodong Wu, Yanli Zhang, Guangzhu Lin

**Affiliations:** a Department of Cardiology, The First Hospital of Jilin University, Changchun, Jilin; b Central Laboratory of the Eastern Division; c Center for Reproductive Medicine, Center for Prenatal Diagnosis; d Echocardiography department, The First Hospital of Jilin University, Changchun, Jilin, China.

**Keywords:** hypertension, left ventricular remodeling, differential expressed genes, bioinformatic analysis, transcription factors

## Abstract

**Introduction:**

Hypertension occurs profoundly in the world, and left ventricular (LV) remodeling containing functional, structural, and mechanical changes induced by uncontrolled blood pressure is a well-known complication, however the underlying mechanism is still obscure.

**Methods:**

To determine differences in gene expression profiles of hypertension and LV remodeling consequence to hypertension, Gene Expression Omnibus 2R online tool was used to identify differently expressed genes. Publicly available databases including GeneMANIA, database for annotation, visualization and integrated discovery, search tool for the retrieva predicting associated transcription factors (TF) from annotated affinities interacting genes, Predicting Associated TF from Annotated Affinities, JASPAR and Comparative Toxicogenomics Database (CTD) were accessed to perform an integrated bioinformatic analysis.

**Results:**

Twenty-one genes (SEC14L3, EML7, PSMD7, PSMA1, GLRX, CNOT10, NBR1, DUSP12, STRAP, SMIM14, RBM8A, TMEM59, TMEM87A,PSMC1, CASP4, ITGB8, DNAJA1, PINK1, PRNP, SAP30L, and EIF3M) were found overexpression in both hypertension and hypertensive LV remodeling. Biological process analysis first revealed that enrichment of these target genes correlated with regulation of cellular amino acid metabolic process, antigen processing and presentation of exogenous peptide antigen via MHC class I, TAP-dependent and proteasome complex, 3 different expression genes (DEGs) participate significantly enriched in NFκB, WNT, and MAPK pathways, meanwhile, 47% DEGs displayed similar co-expression characteristics. Furthermore, the transcription factors associated with key DEGs were identified. Finally, the TF (HAND1, E4BP4, ESR1, VBP, ELK-1, POU3F2) associated with LV remodeling in hypertension were confirmed to act a crucial role in correlated heart diseases.

**Conclusion:**

The present study reveals the targeted genes probably associated with LV remodeling in hypertension by bioinformatics-based analyses, which provides clues for prognosis judgement and pharmacological therapies.

## 1 Introduction

Hypertension is a seriously public health problem all over the world, correlated with most frequent cardiovascular risk factors resulted in heart failure (HF) development and considerable morbidity and mortality.^[1]^ Hypertensive heart disease as the most well-known cardiac complications of arterial hypertension includes increased left ventricular (LV) mass, which often reaches the level of left atrial enlargement and LV hypertrophy (LVH).^[2]^ LV remodeling induced by hypertension can affect ventricular functions, influence survival outcomes, and finally proceed to HF.^[3]^ LV remodeling, especially LVH is an independent predictor of cardiovascular disease events, which is characterized by pathological changes in myocardium on genetic, histological, cellular and molecular level. The mechanisms responsible for LVH progression include both the impacts of cytokines/neurohormones and the response to mechanical stress from higher blood pressure.^[4–6]^ Furthermore, literatures have shown that LVH promote hypertension progression, uncontrolled hypertension accelerates LVH development, whereas the risk of LVH is reduced after blood pressure controlled.^[2,7]^

Electrocardiogram and echocardiography are the most common method to estimate and define LVH, meanwhile cardiac magnetic resonance imaging is brought into clinical studies recently.^[4,8]^ However, there is still lack of consistent criteria for LVH diagnosis in clinic. Finding out more effective diagnostic methods, and fully understanding the correlation in pathogenesis of hypertension and LVH through the association between the 2 diseases can prevent the occurrence of LVH and HF in a more effective manner.^[9]^ The data of gene expression profiles have been increased rapidly in recent years, taking advantage of bioinformatics methods has become a new research hot issue to deeply explore the data of gene expression profiles.^[10,11]^ In this study, bioinformatic methods were used to carry out a series of analysis on gene expression profiles data in patients with hypertension and LV remodeling, and the results were utilized to investigate the bioinformatic significance of the gene expression differences.

## 2 Methods

### 2.1 Microarray gene expression

Profiles of gene expression was explored by using Gene Expression Omnibus (GEO) database (http://www.ncbi.nlm.nih.gov/geo/). Series number GSE71994 and GSE74144 based on platform GPL13497 and GPL6244 were downloaded, respectively. Data of twenty-three controlled hypertensive and seventeen uncontrolled hypertensive patients were selected from GPL13497, while fourteen hypertensive patients with or without left ventricular remodeling were selected from GPL6244 platform, respectively.

### 2.2 Identification of differential expressed genes (DEGs)

Two groups of samples were compared by using GEO2R (https://www.ncbi.nlm.nih.gov/geo/geo2r) to identify DEGs. The results were listed as a table by calculated using the GEO query package R data structures. Then DEGs were identified by *P* < .05, false discovery rate (FDR) < 0.01 (Benjamini and Hochberg's method), and the fold change (FC) was set at 1.2.

### 2.3 Significant modules enrichment analysis

Database for Annotation, Visualization and Integrated Discovery (DAVID, https://david.ncifcrf.gov/tools.jsp) ^[12]^ was a bioinformatics resource to classify DEGs enriched modular functions, cellular components and biological processes, identify enriched pathways correlated with the DEGs. We input the DEGs, selected homo sapiens for Gene Ontology analysis, *P* < .05, Benjamini < .01 in the retrieved results were considered statistical significance.

### 2.4 Analysis by GeneMANIA and protein-protein interaction network

As a flexible, user-friendly web interface for generating hypotheses on gene function, analyzing gene lists, and prioritizing genes for functional assays, GeneMANIA (http://genemania.org/) was used to analyze the interactions among DEGs.^[13]^ Results were exhibited after we imported the gene list of interest in the previous step. Then, we used Retrieval Interacting Genes v11.0 (http://string-db.org/) search tool to provide analysis of interactions among DEG-encoding proteins online.^[14]^

### 2.5 Analysis of transcription factors

We used Predicting Associated TF from Annotated Affinities (http://trap.molgen.mpg.de/pastaa.htm) program, which ranked all TF matrices according to how strongly they associate with your input set, to predict transcription factors (TFs) in the 2 groups.^[15]^ Predicted TFs were shown as we input the DEGs between controlled versus uncontrolled hypertensive patients, and hypertensive patients with versus without LV remodeling. *P* value calculated from hyper geometric distribution was used to evaluate the correlation between the DEGs and TFs, and correlation analysis was performed by TRAP (http://trap.molgen. mpg.de/cgi-bin/home.cgi).^[16]^ Gene sets were uploaded to the database and JASPAR (version 2018, http://jaspar.genereg.net/),^[17]^ a high-quality TF binding profile database to predict DNA binding sites.

### 2.6 Identification of co–DEGs related to LV remodeling or hypertension

We used the comparative toxicogenomics database (http://ctdbase.org/) to find integrated gene-disease, chemical-disease and chemical-gene interactions to predict novel associations and generate expanded networks.^[18]^ Furthermore, these data were analyzed to predict relationships between TFs marker in LV remodeling with hypertension and heart diseases.

### 2.7 Ethical

The exploration was based on the public network database research, ethical approval was not necessary.

## 3 Results

### 3.1 DEGs identification

**Figure 1:**
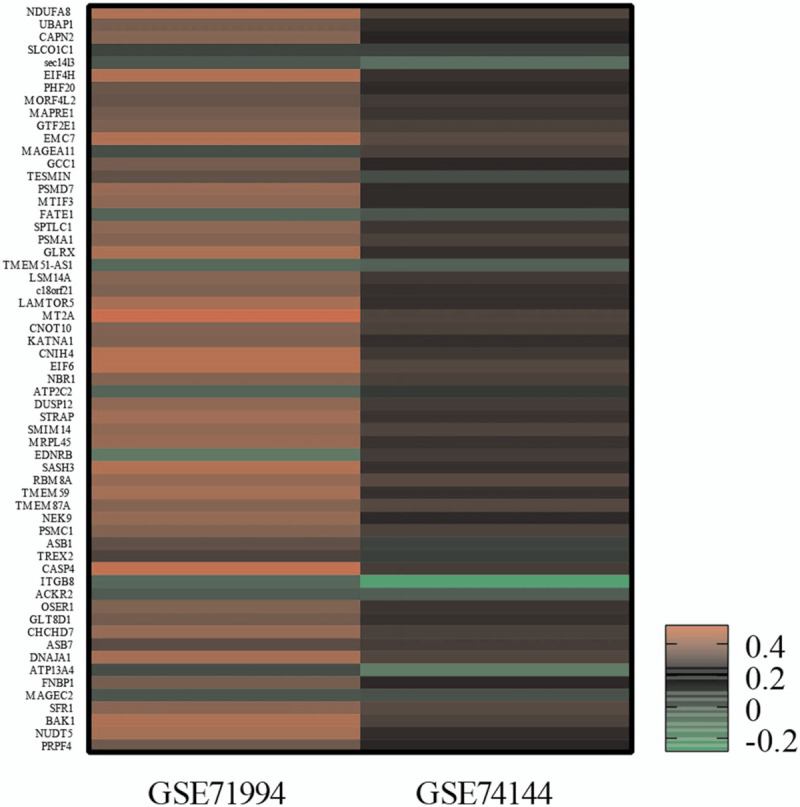
The heat-map of differential expression genes. GSE71994, controlled hypertensive versus uncontrolled hypertensive patients. GSE74144, hypertensive patients with versus without left ventricular remodeling. up-regulated genes were in red and down-regulated genes were in black (*P* < .05).

Compared to the control group, 842 DEGs are identified in GSE71994(including 629 up-regulated genes and 213 down-regulated genes), while the 28232 DEGs in GSE74144(including 13599 up- regulated genes and 14633 down-regulated genes) (Table 1), Figure 1 is the clustering heat-map, and a total of 21 common differential genes were screened from GSE71994 and GSE74144 (Table 2).

**Table 1:**
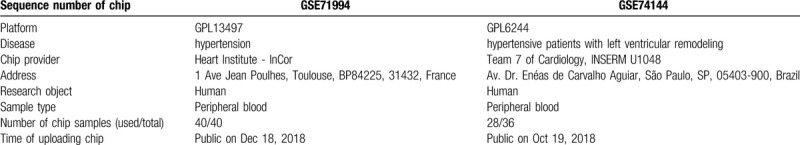
Detailed data of GSE74144 and GSE71994.

**Table 2:**
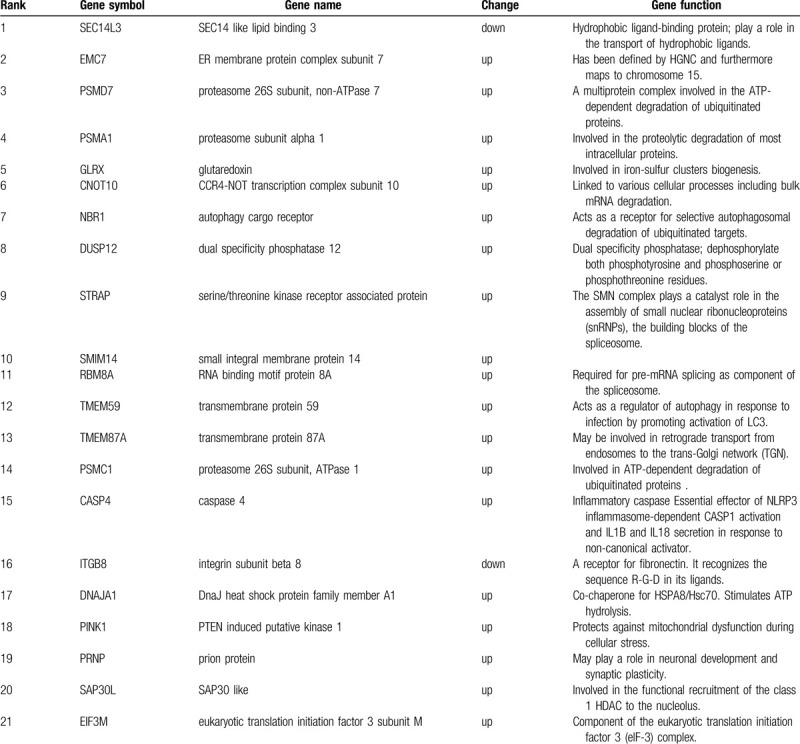
Differential expression genes.

### 3.2 Analysis of functional enrichment

**Figure 2:**
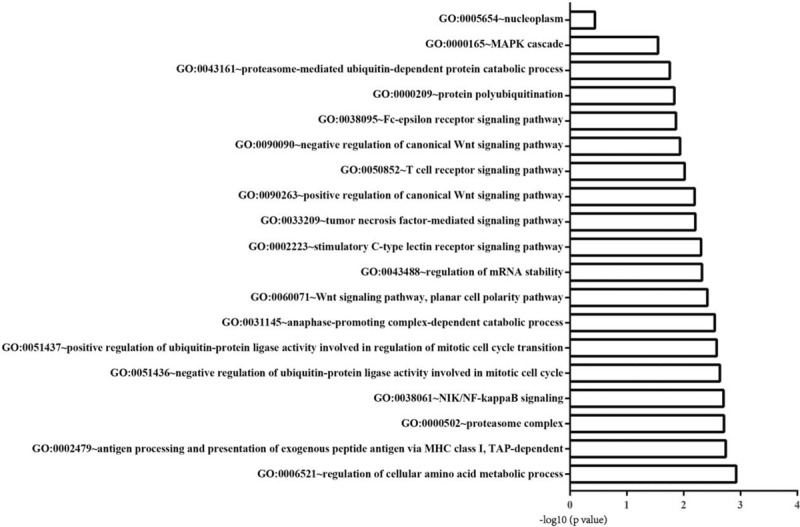
GO enrichment analysis of DEGs between GSE71994 and GSE74144. The y-axis shows significantly enriched Biological Process categories of the targets, and the x-axis shows the enrichment scores of these terms (*P* < .05).

Gene ontology (GO) functional and enrichment analysis are shown in Figure 2. The most enriched GO terms associated with DEGs are regulation of cellular amino acid metabolic process (*P* = .001195), antigen processing and presentation of exogenous peptide antigen via MHC class I, TAP- dependent (*P* = .001817) and proteasome complex (*P* = .001949), The results of functional annotation about pathways PSMA1, PSMC1 and PSMD7 participate in the pathways are significantly enriched in NFκB, WNT, and MAPK pathways.

### 3.3 Analysis by GeneMANIA and analysis of protein-protein interaction (PPI) networks

**Figure 3:**
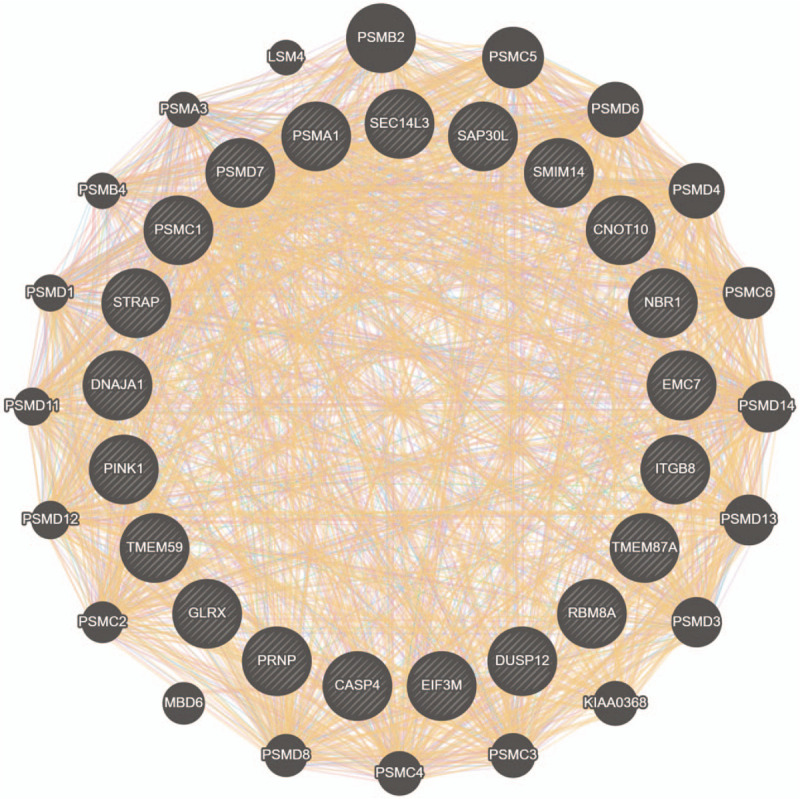
Network of hub genes. Black protein nodes indicate target proteins, and different connecting colors represent different correlations. Functional association of targets was analyzed by using GeneMANIA. Violet lines represent co-expression between these genes, yellow lines represent predicted between these genes, and red lines represent physical interactions.

**Figure 4:**
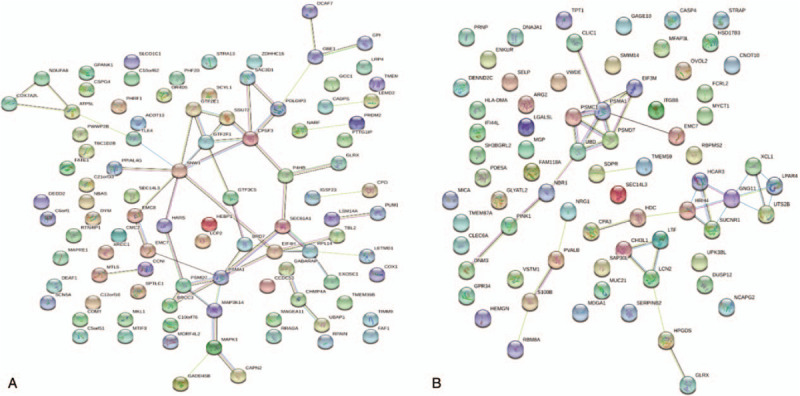
PPI network analysis of DEGs. (A) PPI network analysis of DEGs in GSE74144. (B) PPI network analysis of DEGs in GSE71994. Circle represents gene; line represents PPI between genes, and results inside the circle represent protein structure. Line colors stand for the evidence of PPI.

Of twenty-one targets and their interacting proteins, the result showed that 47% displayed similar co-expression characteristics, 34.71% were predicted and 10.13% had physical interactions, which were presented in Figure 3. Then, the top 100 genes of each database were updated to search tool for the retrieval interacting genes to construct the PPI network, PSMD7, PSMC1, EIF3M, PSMA1 and EMC7 are highlighted, and is shown in Figure 4.

### 3.4 TFs analysis

**Figure 5:**
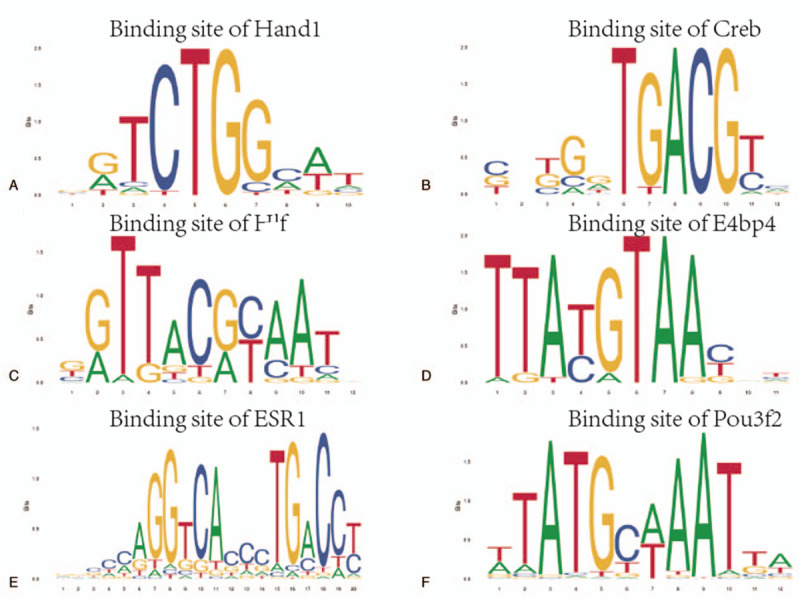
TFs binding site predicted by JASPAR. TFs = transcription factors.

**Figure 6:**
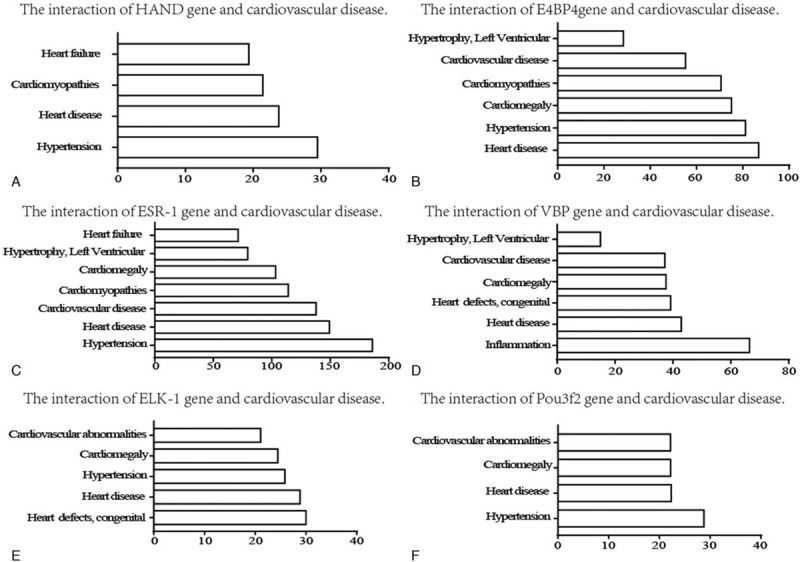
Relationship between TFs and heart diseases based on the CTD database. CTD = comparative toxicogenomics database, TFs = transcription factors.

TFs that modulate gene expression in hypertension and hypertensive patients with LV remodeling predicted by predicting associated TF from annotated affinities were exhibited in Table 3. As shown in Figure 5, TFs-binding sites were predicted by JASPAR. The comparative toxicogenomics database database showed that the TFs associated with LV remodeling in hypertension acted a crucial role in correlated heart diseases, these data were shown in Figure 6.

**Table 3:**
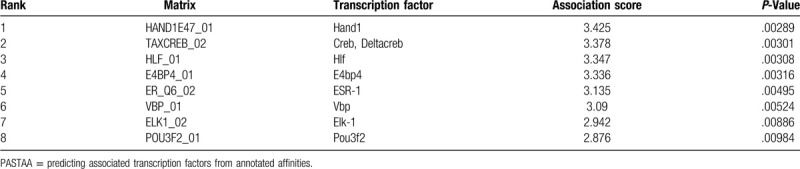
Results of PASTAA analysis.

## 4 Discussion

In brief, LVH is a complication of hypertension, LVH regression can reduce subsequent incidence rate of cardiovascular disease events. However, mechanistic links between hypertension treatment and LVH regression are not well understood, and the correlation at the genetic level between hypertension and LV remolding has attracted more and more attention recently. Biomarkers for LV remodeling after hypertension is limited to electrocardiogram and echocardiography.^[19]^ Novel approaches that identify the potential risk factor of progressive LV remodeling in high risk individuals, with more manual intervention and surveillance my help reduce occurrence rate. Nowadays, microarray analysis was used to help defining an earlier diagnosis and lower misdiagnosis rate,^[20]^ the application of microarray analysis has achieved considerable bioprocesses associated with hypertension. Previous studies had reported that ion channel-associated gene such as PRELP, CLIC2, SCN2B, COL1A1-2, COMP and KCNJ5 play important roles in related bioprocesses.^[21–23]^ Purpose of this article is to find the HUB genes between hypertension and LV remodeling, underlining regulation effects by TFs on differential gene expression. The validity of target gene sets was verified by reference to the existing databases.

By comparing DEGs in hypertensive and uncontrolled hypertensive patients with hypertensive patients with or without LV remodeling samples, we predict that SEC14L3, EML7, PSMD7, PSMA1, GLRX, CNOT10, NBR1, DUSP12, STRAP, SMIM14, RBM8A, TMEM59, TMEM87A,PSMC1, CASP4, ITGB8, DNAJA1, PINK1, PRNP, SAP30L, and EIF3M may play roles in hypertension development. The study is aimed to explore the mechanism underlying the level of differential expression.

DEGs of hypertension exhibit up-regulation in GO terms of extracellular matrix and focal adhesion, autophagy, and proteasome-mediated ubiquitin-dependent protein catabolic process as previously reported.^[24]^ Recent studies have revealed a link between autophagy and pathophysiological left ventricular remodeling due to stress overload.^[25]^ During adaptive remodeling of LVH, compensatory increases in protein synthesis lead to the accumulation of toxic misfolded molecules and protein aggregates. Autophagy is the primary cellular mechanism for removing these toxic protein aggregates and dysfunctional organelles in order to maintain cardiac integrity.^[26,27]^ In our study, NBR1 and RBM8A genes are predicted as a receptor for selective autophagosome degradation of ubiquitinated targets. Although at the early stage of RVH, overall cardiac function was relatively retained, its biological effects were highlighted by altered bioenergetic metabolism, including elevated cellular apoptosis, autophagy, and mitochondrial degradation signals, and hindered mitochondrial respiratory-chain subunit proteins production.^[28]^ In the present study, PSMD7, PSMC1, DNAJA1 and PINK1 are all up-regulated, which probably lead to hypertension progression and left ventricular remodeling through ATP-dependent degradation of ubiquitinated proteins or phosphorylating mitochondrial proteins. In a variety of pathologic factors that may be activated during adaptive LVH to LV remodeling, the qualitative and quantitative changes of cardiomyocyte extracellular matrix (ECM) may be the key factors causing the change of cardiomyocyte arrangement.^[29]^ PSMA1 encodes a non-collagen protein in ECM; and high expression of PSMA1 leads to cardiomyocyte apoptosis and myofilaments loss.^[30]^ eIF3 is revealed to play a role in regulating the translation of mRNA subsets and in regulating cell cycle progression and cell proliferation.^[31]^ Furthermore, it has also been shown that in lungs of pulmonary fibrosis eIF3 expression of was significantly elevated ^[32]^ and renal fibroblasts ^[33]^ associated with exacerbated accumulation of deposition of the ECM.

DEGs may affect TFs to promote hypertension progression or accelerate the occurrence of complications. HAND1 plays important roles in both cardiac morphogenesis and trophoblast-giant cells differentiation.^[34]^ It may also affect septal defects in the human heart, thereby playing wider roles in human congenital heart diseases.^[35]^ CREB, significantly increased in LVH,^[36]^ not only contributing to the primary modulating factors of the endoplasmic reticulum stress regulating, but also stimulating transcription upon binding to the DNA cAMP response element.^[37]^ ESR1 is an estrogen nuclear receptor; expressed in wide ranges of cells and tissues, such as endothelial cells and smooth muscle from vessels.^[38–40]^ The exposure of estrogen is reported to be associated with increase vasodilatation and cardiovascular system against ROS-mediated cellular injury protection.^[41]^ The expression level of ESR1 is positively correlated with the occurrence rate of cardiovascular diseases, especially hypertension differs between males and females.^[42]^ Mount of evidence suggests that in postmenopausal women, estrogen deficiency plays major roles in the pathogenesis of cardiovascular diseases.^[43]^ Our findings provided basis for further exploring the application of gene target as a novel hypertension treatment.

## 5 Conclusions

We used bioinformatics analysis to reveal the target genes that may be related to hypertension and LV remodeling, providing clues for early diagnosis and target therapy. However, the predicted target genes still need be verified in the further studies for clinical application.

## Acknowledgments

We appreciated critical comments and invaluable suggestions from Dr. Huanfa Yi's (Central Laboratory, The First Hospital of Jilin University) on this manuscript.

## Author contributions

**Data curation:** Yanli Zhang.

**Formal analysis and Software:** Bo Pang.

**Methodology:** Guodong Wu.

**Project administration & Supervision:** Guangzhu Lin.

**Writing – original draft:** Bo Pang.

**Writing – review & editing:** Cong Hu.
